# Lidocaine and Bupivacaine Downregulate MYB and *DANCR* lncRNA by Upregulating *miR-187-5p* in MCF-7 Cells

**DOI:** 10.3389/fmed.2021.732817

**Published:** 2022-01-13

**Authors:** Chiao-Yi Lin, Wen-Ting Tseng, Yao-Yin Chang, Mong-Hsun Tsai, Eric Y. Chuang, Tzu-Pin Lu, Liang-Chuan Lai

**Affiliations:** ^1^Graduate Institute of Physiology, College of Medicine, National Taiwan University, Taipei, Taiwan; ^2^Department of Anesthesiology, Hsinchu MacKay Memorial Hospital, Hsinchu, Taiwan; ^3^Department of Electrical Engineering, Graduate Institute of Biomedical Electronics and Bioinformatics, National Taiwan University, Taipei, Taiwan; ^4^Graduate Institute of Biotechnology, College of Bioresources and Agriculture, National Taiwan University, Taipei, Taiwan; ^5^Bioinformatics and Biostatistics Core, Center of Genomic and Precision Medicine, National Taiwan University, Taipei, Taiwan; ^6^Graduate Institute of Biomedical Electronics and Bioinformatics, College of Electrical Engineering and Computer Science, National Taiwan University, Taipei, Taiwan; ^7^College of Biomedical Engineering, China Medical University, Taichung, Taiwan; ^8^Graduate Institute of Epidemiology and Preventive Medicine, College of Public Health, National Taiwan University, Taipei, Taiwan

**Keywords:** *miR-187-5p*, *MYB*, *DANCR*, bupivacaine, lidocaine

## Abstract

**Background:** Breast cancer is the most common malignancy and a leading cause of death among women. The majority of patients require surgery, and retrospective studies have revealed an association between anaesthetic techniques during surgery and clinical outcomes. Local anaesthetics (LAs) influence carcinogenesis by interacting with non-coding RNAs (ncRNAs). However, the detailed mechanisms underlying the association between LAs and ncRNAs remain unclear.

**Methods:** In this study, the effects of two commonly used LAs, lidocaine and bupivacaine, on the malignancy of MCF-7 breast cancer cells were investigated. The expression profiles of the microRNAs (miRNAs) that responded to treatment with LAs were determined through next-generation sequencing.

**Results:** Data from the functional assay revealed that the LAs suppressed the proliferation of MCF-7 cells. The result of next-generation sequencing revealed that 131 miRNAs were upregulated, following treatment with the LAs. Validation using polymerase chain reaction (PCR) identified *miR-187-5p* as a potential biomarker, and it was selected for further analyses. Prediction with bioinformatics tools and luciferase reporter assays revealed that *MYB* is a direct target gene of *miR-187-5p*. Based on the hypothesis that lncRNAs acts as miRNA sponges, the target lncRNA, *DANCR*, of *miR-187-5p* was predicted using DIANA-LncBase v2 and validated using luciferase reporter assays. In addition, the reciprocal suppressive effect between *DANCR* and *miR-187-5p* was determined.

**Conclusions:** This study suggests that one of the anti-tumour mechanisms of lidocaine and bupivacaine is mediated through the *DANCR-miR-187-5p-MYB* axis. This may provide a novel molecular mechanism of tumour suppression in breast cancer.

## Introduction

Breast cancer is one of the most common malignancies and a leading cause of death among women (https://gco.iarc.fr/). Most patients require surgery; however, residual disease from scattered micro-metastases and tumour cells related to surgery is usually inevitable (1). Experimental and clinical retrospective studies indicate an association between the types of anaesthetic techniques used during the cancer resection surgery and outcomes (1, 2). There are conflicting results on the administration of volatile anaesthetics and opioids and cancer development, metastasis, and recurrence (3, 4). Regional anaesthesia is hypothesised to attenuate immunosuppression and surgical stress by minimising the requirement for opioids and volatile anaesthetics, therefore improving the long-term postoperative outcomes (2). The overall survival for different local anaesthetics (LAs) varies among the clinical studies; however, laboratory studies suggest that LAs have direct inhibitory effects on tumour cells (5, 6).

Lidocaine and bupivacaine are commonly used amide-type LAs for regional anaesthesia and peripheral nerve blockade, providing excellent perioperative pain relief, especially in breast cancer surgery (7). The mechanisms underlying the inhibitory effect of LAs on cancer cell proliferation, migration, and metastasis, are elucidated (5, 8, 9). However, the precise mechanism remains unclear; it could involve sodium channel blockade, DNA demethylation (10, 11), or interactions between LAs and non-coding RNAs (ncRNAs). Lidocaine inhibits the growth and invasion of gastric carcinoma cells by upregulating miR-145 (12). It inhibits proliferation and induces apoptosis in colorectal cancer cells by upregulating miR-520a-3p and targeting EGFR (13).

Dysregulated gene expression is a major hallmark of cancer. ncRNAs play a critical role in tumourigenesis, growth, and progression (14–16). NcRNAs are categorised as small ncRNAs (sncRNAs) and long ncRNAs (lncRNAs) with a cut-off length of 200 bp; they are directly or indirectly involved in diverse biological processes through epigenetic, transcriptional, and post-transcriptional mechanisms (17). MicroRNAs (miRNAs) are a type of short ncRNAs that regulate cellular proliferation, differentiation, apoptosis, adhesion, epithelial-mesenchymal transition (EMT), and metastasis in various cancers (18). The competing endogenous RNA (ceRNA) theory (19) states that miRNAs recognise miRNA response elements (MREs) in different RNA molecules, and induce target repression through miRNA-RNA-induced silencing complex (RISC)-mediated degradation. Therefore, the transcriptional regulation of diverse RNAs, including mRNAs and ncRNAs, can regulate biological processes through a novel MRE-mediated mechanism (19).

The tumour-suppressive effects of lidocaine and bupivacaine were studied in MCF-7 cells. The effects of these LAs on the function of *miR-187-5p*, Differentiation Antagonising Non-Protein Coding RNA (*DANCR)*, and *MYB* were investigated for elucidating the underlying mechanism. Our findings provide novel insights into the process of tumour suppression for future breast cancer therapy.

## Methods

### Cell Culture

MCF-7 cells were purchased from Bioresource Collection and Research Center (Hsinchu, Taiwan), and HEK-293T cells were provided by Dr. Shau-Ping Lin (Institute of Biotechnology, National Taiwan University, Taiwan). All cells were cultured in Dulbecco's Modified Eagle Medium (DMEM) (Gibco, Thermo Fisher, CA, USA) containing 1% penicillin-streptomycin (Gibco) and 10% foetal bovine serum (FBS) (HyClone, Logan, UT, USA). The culture plates were maintained at 37°C in a humidified atmosphere of 5% CO_2_ and were routinely tested for Mycoplasma sp. using Mycoplasma polymerase chain reaction (PCR) Detection Kit (ABM Inc., Vancouver, BC, Canada).

### Cell Viability Assay

MCF-7 cells were seeded at 5 ×10^3^ cell into 96-well plates 1 day before lidocaine or bupivacaine (Sigma, St. Louis, MO, USA) treatment at the indicated concentrations. LAs and the controls were diluted in 200 μL DMEM for each well. For the thiazolyl blue tetrazolium bromide (MTT) colorimetric assay, 5 mg/mL of MTT (Sigma) were added to each well and incubated at 37°C for 4 h. The supernatants were removed and replaced with dimethyl sulfoxide (DMSO) (Sigma). The absorbance was measured using a microtiter plate reader (BioTek, Winooski, VT, USA) at 570 nm.

### Wound Healing Assays

MCF-7 cells were seeded at a density of 2.5 ×10^4^ cells/well in a medium containing 10% FBS and incubated overnight. The culture inserts were removed and an image of the gap area at 0 h was captured. The cells were further incubated at 37°C in a 5% CO_2_ incubator, and the images were captured at 12, 24, and 36 h, respectively. The cell migration ability in the gap area was quantified using ImageJ v1.8.0 (National Institutes of Health, USA).

### Cell Migration

Migration assays were performed using SPLInsert^TM^ Hanging plate (SPL Life Sciences, Pocheon, South Korea). The upper chamber of the transwell unit was seeded at 4 ×10^4^ cells/well in 0.2 mL serum-free DMEM. The lower chambers were loaded with 0.6 mL DMEM containing 10% FBS as chemo-attractant. The MCF-7 cells were incubated at 37°C for 36 h. A methanol: acetic acid (3:1) mixture was added to the lower chamber for fixing the cells by incubating at 25°C for 10 min. The non-migrating cells on the inner transwell membrane were removed carefully, and the membranes were stained with 1 mL of 0.5% crystal violet. The stained cells were solubilized with 10% acetic acid, imaged, and analysed using ImageJ v1.8.0.

### Western Blotting

The proteins were extracted from the cells lysed with radioimmunoprecipitation assay buffer (RIPA) Lysis Buffer (EMD Millipore, Billerica, MA, USA) and the concentration was determined using Coomassie Protein Assay Reagent (Thermo Fisher, Waltham, MA, USA). The extracted protein samples (20 μg) were separated using 10% sodium dodecyl sulphate–polyacrylamide gel electrophoresis (SDS-PAGE) and transferred to polyvinylidene fluoride (PVDF) membranes (GE healthcare, Chicago, IL, USA). The membranes were incubated with primary rabbit polyclonal antibodies against c-MYB (Proteintech, Rosemont, IL, USA) and β-actin (GeneTex, Irvine, CA, USA), at 4°C overnight; the samples were hybridised with horseradish peroxidase (HRP) conjugated anti-rabbit IgG (GeneTex) at 25°C for 1 h. The blotted proteins were detected using an enhanced chemiluminescence (ECL) system (Millipore, Billerica, MA, USA) equipped with a BioSpectrum Imaging System (UVP, Upland, CA, USA). The images were analysed using ImageJ v1.8.0.

### RNA Extraction, Reverse Transcription, and Quantitative Real Time Polymerase Chain Reaction

The RNA was extracted using TRIZOL (Invitrogen, Carlsbad, CA, USA) and reverse-transcribed using High-Capacity cDNA Reverse Transcription Kit (Applied Biosystems, Carlsbad, CA, USA). The RNA quality was detected by a spectrophotometer (NanoDrop; Thermo Scientific) with the A260/A280 ratio between 1.8~2.0. Samples were kept in RNase free water buffer at neutral pH. All RNA samples were stored at −80°C. One μg of total RNA was reverse-transcribed by High-Capacity cDNA Reverse Transcription Kit (Applied Biosystems, Carlsbad, CA, USA). SuperScript IV Reverse Transcriptase (Invitrogen) was used for reverse transcription of the miRNAs in accordance with the manufacturer's instructions. Two point five percent of each reaction was used as template for quantitative PCR with OmicsGreen qPCR MasterMix (OmicsBio, New Taipei City, Taiwan) and the reactions were performed on StepOnePlus Real-Time PCR System (Thermo Fisher, Waltham, MA, USA). The primers were checked for only generating a single melting curve peak and detailed information are provided in Supplementary Table 1. The relative expression was normalised to that of 18S rRNA or U6 snRNA, using the 2^−Δ*ΔCt*^ method.

### Library Preparation and Sequencing

The total RNA was extracted using TRIZOL reagent (Invitrogen). The small RNA library was constructed from total RNA (2 μg) using the TruSeq Small RNA Library Prep Kit (Illumina Inc., San Diego, CA, USA), according to the manufacturer's instructions. A total of 75 single-read nucleotides were obtained from each miRNA library using NextSeq500 (Illumina Inc., CIC bioGUNE, Bilbao, Spain). The sequencing data were submitted to the Gene Expression Omnibus (GEO) (accession number: GSE171282).

### Sequencing Data Analyses

The raw Illumina FASTQ sequencing reads were mapped to the miRBase mature miRNA reference (http://www.mirbase.org/) using Partek Flow™ v5.0 (Partek Inc., St. Louis, MO, USA) with Bowtie alignment algorithm. The Bowtie alignment method was generally thought to be optimal for sequences less than 50 bp. The adapter sequences were first removed from each FASTQ reads, and the remaining bases were trimmed from the 3′ end with a minimum Phred quality score of 20. A minimum read-length philtre of 15-bases in length was used in this work. A minimum seed length of 10 that was consistent with the standard setting provided by the Partek Flow pipeline was chosen for the Bowtie aligner. The remaining reads were aligned to the human genome reference (RefSeq Hg19). Raw miRNA expression reads were normalised by scale normalisation and then processed by log_2_ transformation. Then, miRNAs with extremely low expression (log_2_ <5 in all samples) were removed from further analysis. Then, differentially expressed miRNA profiles between groups were identified by log_2_ fold-change value ≧2X or < -1X and P-value <0.05 using a two-sided Student's t-test. Principal component analysis using the expression values of total miRNAs was used to visualise the similarity of different groups.

### Transfection

MCF-7 cells were cultured in an antibiotic-free medium to 70–80% confluence in 6-well plates (2.5 ×10^5^ cells/well) and transfected with the 0.025 nmol of *miR-187-5p* mimic (GenePharma, Shanghai, China) or negative control (GenePharma), using Lipofectamine 2000 as the transfection reagent (Invitrogen), following the manufacturer's instructions.

The MCF-7 cells were transfected with 0.5 nmol of pcDNA-*DANCR* or the empty vector (Dharmacon, New Taipei city, Taiwan) using jetPRIME (Polyplus-transfection, New York, NY, USA) reagent, following the manufacturer's instructions. After 4 h, the medium was replaced with fresh medium containing serum. The cellular RNA expression after 48 h was determined using qRT-PCR.

### Plasmid Construct and Site-Directed Mutagenesis

To determine promoter activity by luciferase assay, the luciferase expression plasmid pMIR-REPORT-*c-MYB 3*′*UTR* and pMIR-REPORT-*DANCR* was purchased from the BioMed Resource Core of the 1st Core Facility Lab, National Taiwan University College of Medicine (Taipei, Taiwan). Briefly, *c-MYB 3*′*UTR* region encompassing 2,493–3,684 bp relative to the transcription start site was amplified from the human genomic DNA by PCR. The *c-MYB 3*′*UTR* was subcloned into pMIR-REPORT vector, and the final construct was called pMIR-REPORT-*c-MYB 3*′*UTR*. *DANCR* encompassing 1–915 bp relative to the transcription start site was amplified from the human genomic DNA by PCR and the final construct was called pMIR-REPORT-*DANCR*.

*miR-187-5p* binding sites of *c-MYB 3*′*UTR* were located at 2,710–2,734 and 3,634–3,656 bp. Besides, *miR-187-5p* binding sites of *DANCR* were located at 134–157 and 380–408 bp. To determine binding activity of *miR-187-5p* by luciferase assay, the luciferase expression plasmid pMIR-REPORT-*c-MYB 3*′*UTR* S1, pMIR-REPORT-*c-MYB 3*′*UTR* S2, pMIR-REPORT-c-MYB 3′UTR S12, pMIR-REPORT-*DANCR* S1, pMIR-REPORT-*DANCR* S2, and pMIR-REPORT-DANCR S12 was purchased from the BioMed Resource Core of the 1st Core Facility Lab, NTU-CM (Taipei, Taiwan). In addition, the mutated sequences were validated by sequencing.

### Luciferase Reporter Assay

Luciferase assays were performed with HEK-293T cells using the Dual-Glo® luciferase reporter assay system (Promega, Fitchburg, WI, USA). Cells (5 ×10^4^/well) were transfected with 100 ng reporter constructs containing the wild-type or mutant-type of *MYB*, the wild-type or mutant-type of *DANCR* and 2 ng Ranilla luciferase plasmid (BioMed Resource Core of the 1st Core Facility Lab, National Taiwan University College of Medicine, Taiwan), using the jetPRIME (Polyplus-transfection) reagent. The cells were additionally transfected with 0.025 nmol of *miR-187-5p* (GenePharma). After 24 h, the cells were lysed and the Renilla luciferase signals were used for normalisation, according to manufacturer's protocol.

### Statistical Analyses

The experimental data are presented as the mean ± standard deviation (SD) of at least three independent experiments. The genes with statistically significant expression patterns in the different samples were determined by a two-tailed Student's *t*-test using GraphPad Prism v5 (GraphPad Software, Inc., La Jolla, CA, USA). *p*-values < 0.05 were considered statistically significant.

## Results

### LAs Significantly Suppressed Cell Viability and Migration

**Figure 1:**
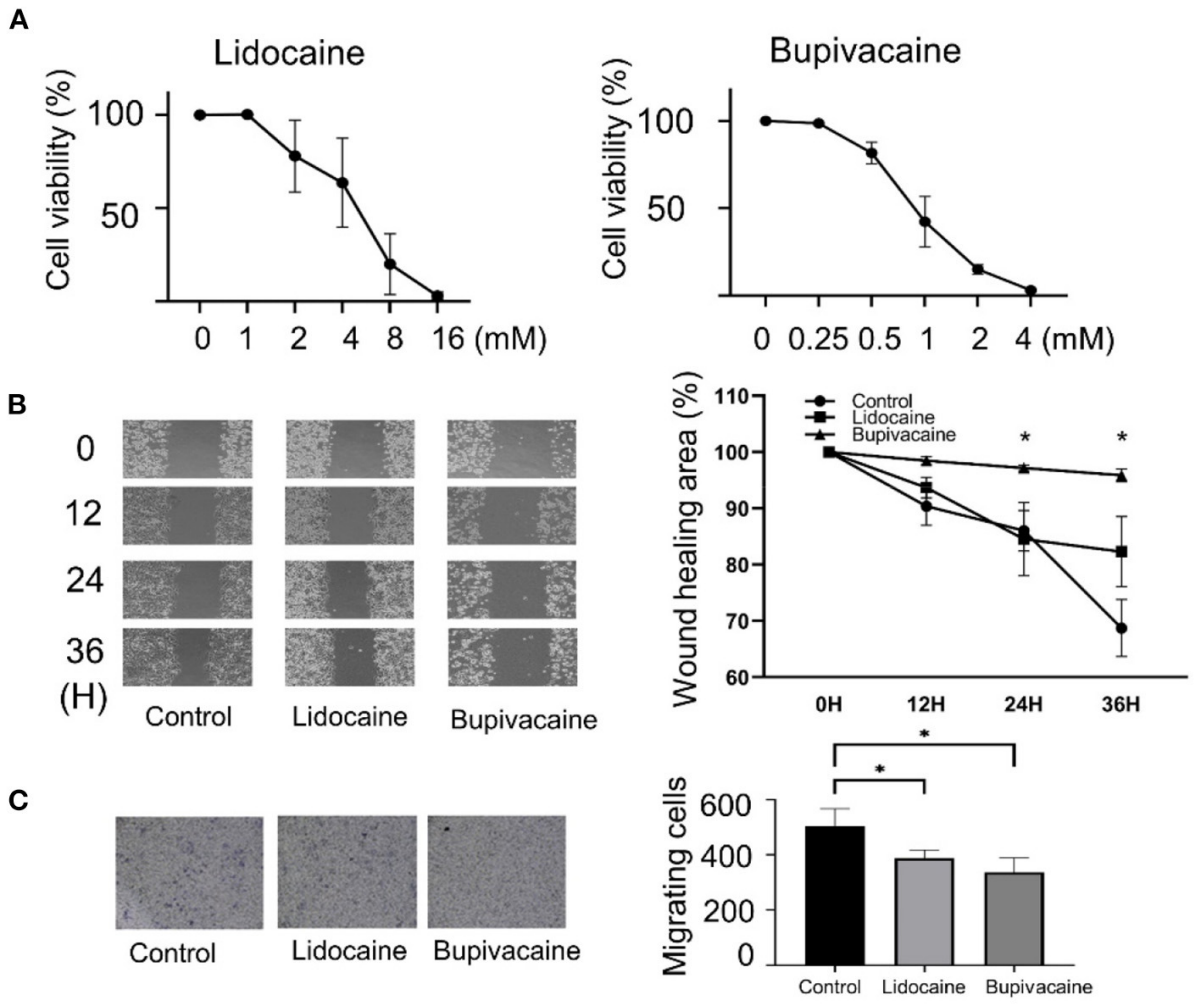
Lidocaine and bupivacaine inhibited the proliferation and migration of MCF-7 cells. **(A)** Cell proliferation determined using the MTT assay. The growth of MCF-7 cells measured after 6 h of treatment with serially diluted concentrations of lidocaine or bupivacaine. The proliferation rate was normalised to that at 0 h. **(B)** Wound healing assay. Left: Images after 0, 12, 24, and 36 h of treatment with lidocaine (4 mM) or bupivacaine (1 mM). Right: The migratory ability quantified using the reduction in wound size over time by ImageJ software v1.8.0. **(C)** Transwell migration assay. Right: The cells were seeded after 24 h of treatment with lidocaine (4 mM) or bupivacaine (1 mM). Left: Cell migration measured after 36 h of seeding. All data are presented as mean ± SD (*n* = 3). *p < 0.05.

To determine the effects of lidocaine and bupivacaine on MCF-7 cells, the cell viability following treatment with different concentrations of lidocaine or bupivacaine for 6 h was determined. Lidocaine and bupivacaine inhibited the growth of MCF-7 cells in a dose-dependent manner (Figure 1A). The median lethal dose (LD_50_) of lidocaine (4 mM) and bupivacaine (1 mM) were used as the concentrations for the following experiments, respectively. Wound healing and transwell invasion assays were performed using MCF-7 cells treated with lidocaine or bupivacaine for 12, 24, and 36 h. Both the LAs significantly reduced the migration and invasive ability of MCF-7 cells (Figures 1B,C), suggesting suppression of malignancy in the MCF-7 cells.

### Differential miRNA Expression Profiles Following LA Treatment

**Figure 2:**
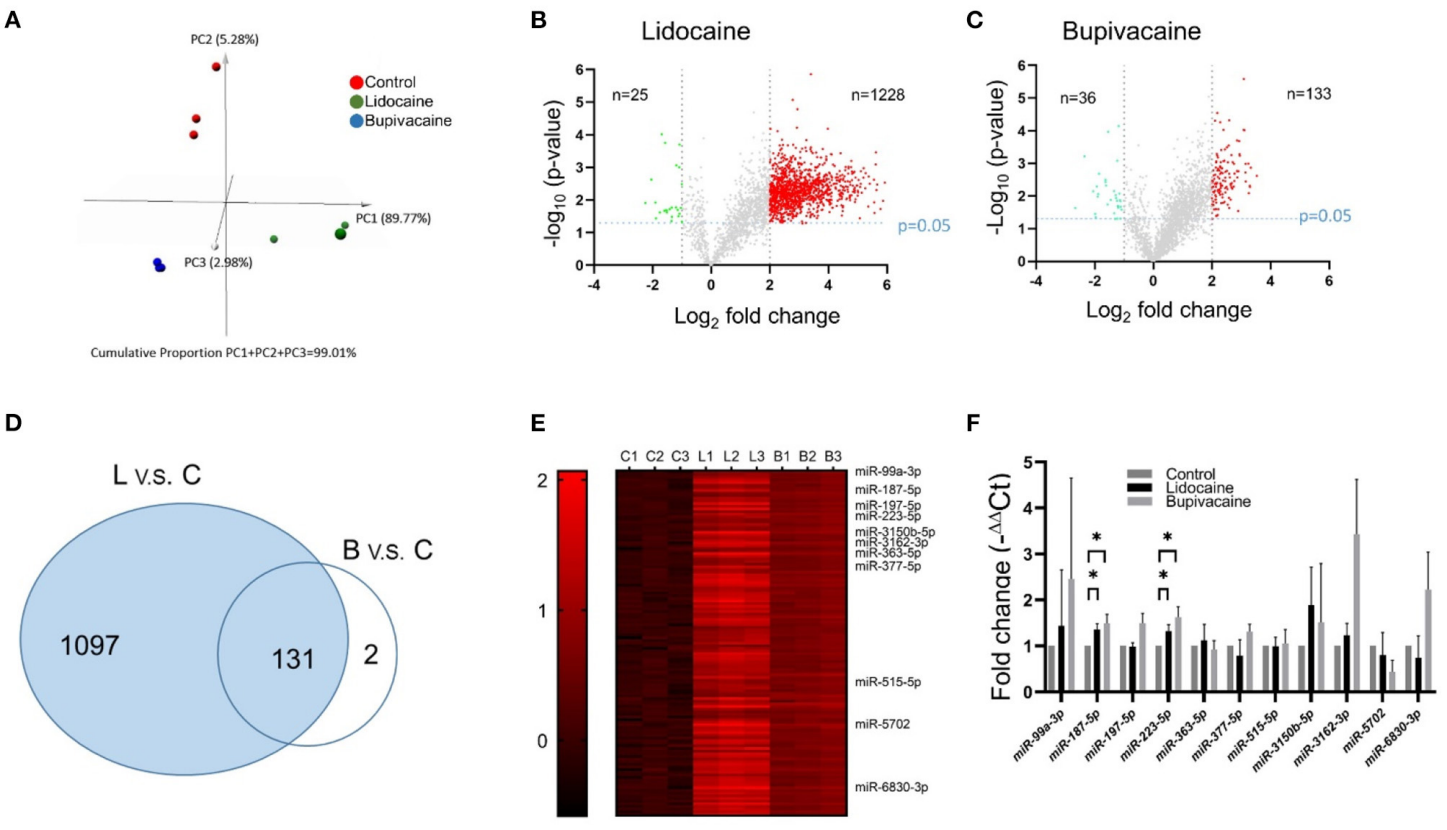
Identification of miRNAs in MCF-7 cells that responded to treatment with lidocaine or bupivacaine. **(A)** PCA of differentially expressed miRNAs in response to treatment with lidocaine (4 mM) or bupivacaine (1 mM). **(B,C)** Volcano plot of the differentially expressed miRNAs in response to treatment with lidocaine **(B)** or bupivacaine **(C)** determined by next-generation sequencing. **(D)** Venn diagram of the differentially expressed miRNAs. The expression of 131 miRNAs was upregulated following treatment with lidocaine or bupivacaine. **(E)** Heat map of the upregulated miRNAs (*n* = 131) that responded to treatment with both lidocaine and bupivacaine. **(F)** PCR validation of the selective differentially expressed miRNAs in MCF-7 cells, following treatment with lidocaine and bupivacaine. The expression levels measured using qRT-PCR and normalised to that of U6 snRNA. L, lidocaine; B, bupivacaine; C, control. All data are presented as mean ± SD (*n* = 3). *p < 0.05.

To identify the miRNAs that mediated the effects of the LAs on MCF-7 cells, next-generation sequencing was used for identifying the differentially expressed miRNAs, following lidocaine or bupivacaine treatment. Principal component analysis (PCA) was performed for the samples (lidocaine, bupivacaine, and control groups, nine dots in total) using the data for 2,522 differentially expressed miRNAs in the three groups (Figure 2A). The miRNAs in the same group showed similar expression profiles, while the expression profiles in the lidocaine- and bupivacaine-treated groups were distinct from those of the control.

The miRNAs that were differentially expressed in the lidocaine- and bupivacaine-treated groups were filtered using the criteria: fold change ≥ 2.0X and *p*-value ≤ 0.05. The expression of 1,228 and 133 miRNAs was significantly upregulated in the lidocaine (Figure 2B) and bupivacaine groups (Figure 2C), respectively (red dots). The expression of 25 and 36 miRNAs was significantly downregulated (fold change < -1X and *p*-value ≤ 0.05) in the lidocaine (Figure 2B) and bupivacaine groups (Figure 2C), respectively (green dots). The genes that were not differentially expressed are depicted in grey. Among the upregulated miRNAs, 131 were common to both the lidocaine and bupivacaine groups (Figure 2D). The number of downregulated miRNAs common to both the groups was low; therefore, only the upregulated miRNAs were selected for subsequent studies. The expression of the selected miRNAs is depicted using a heatmap (Figure 2E).

The data from next-generation sequencing was validated by selecting the miRNAs whose expression was significantly higher and for which experimentally validated data were available. The expression of only *miR-187-5p* and *miR-223-5p* was significantly (*p* < 0.05) higher than that of the control. The expression of the other miRNAs examined using qRT-PCR was not statistically different between the lidocaine and bupivacaine groups (Figure 2F).

### LAs Downregulated MYB Expression via *miR-187-5p*

To identify the genes regulated by *miR-187-5p* and ascertain their functional relevance, we used the TargetScan tool (http://www.targetscan.org/vert_72/) for identifying the downstream target genes. The *MYB* transcription factor, a proto-oncogene, was a predicted target gene of *miR-187-5p*. *In vitro* and *in vivo* studies indicated that *MYB* plays an important role in different cancers. *MYB* overexpression and dysregulation is observed in almost all breast tumours, and is associated with poor prognosis (20, 21). *MYB* also regulates cell proliferation, differentiation, and angiogenesis (22), and therefore, was selected for further studies.

**Figure 3:**
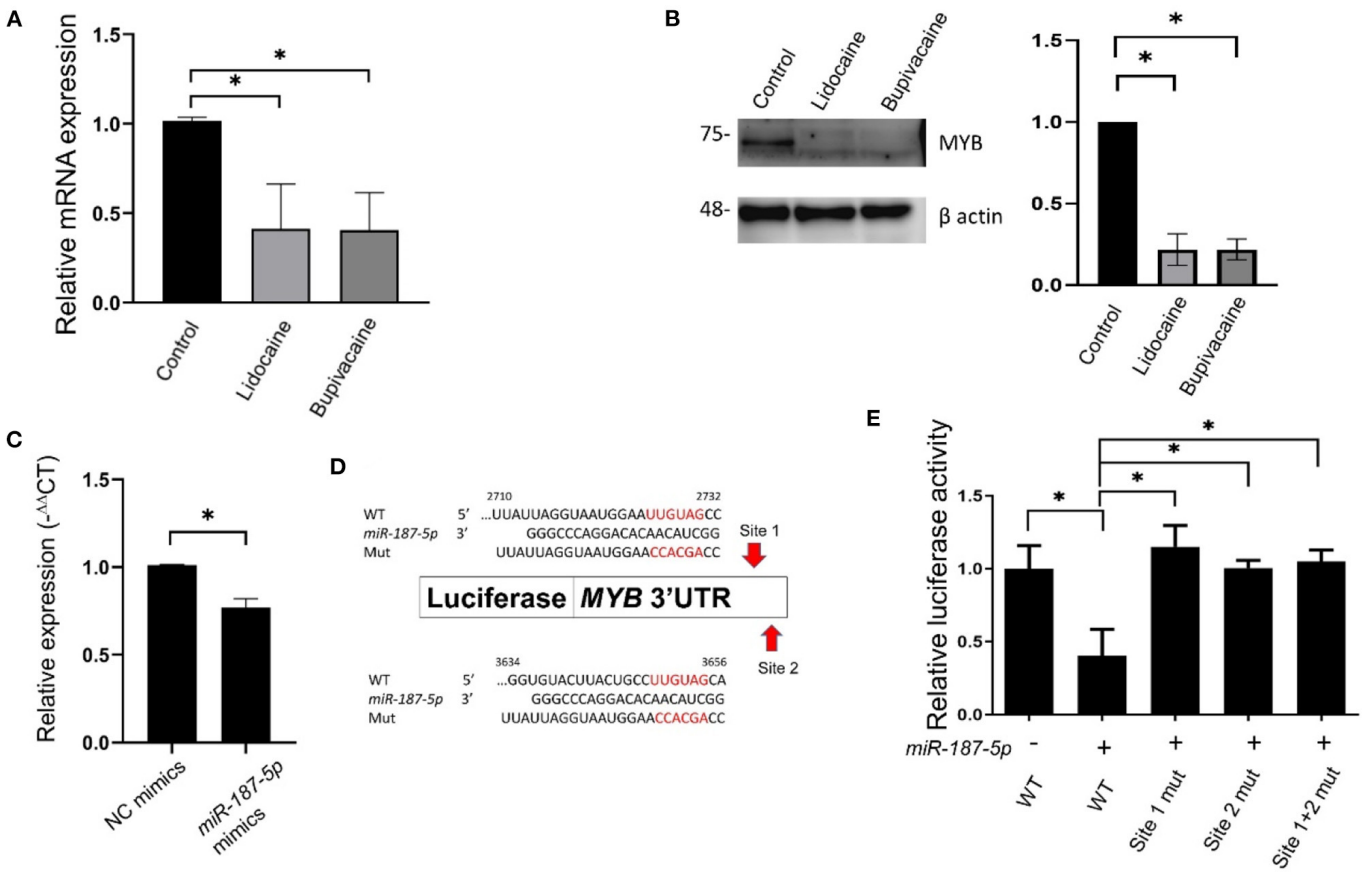
Downregulation of *MYB* in MCF-7 cells following lidocaine and bupivacaine treatment. **(A)** The relative mRNA levels of *MYB* in MCF-7 cells following treatment with lidocaine (4 mM) or bupivacaine (1 mM). The expression levels of *MYB* were measured using qRT-PCR and normalised to that of 18S rRNA. **(B)** Western blots of MYB in MCF-7 cells following treatment with the LAs. Right: graphical representation of the results of western blotting. Left: quantification of the western blots. β actin was used as the loading control. **(C)** Relative expression levels of *MYB* in MCF-7 cells overexpressing *miR-187-5p*. The cells were transfected with 2 μg of *miR-187-5p* or control mimics. The expression levels were detected using qRT-PCR and normalised to that of 18S rRNA. **(D)** Schematic diagram of the putative binding site of *miR-187-5p* in the 3′-UTR region (2,493 ~ 3,684 bp) of *MYB*. **(E)** Luciferase reporter assays of *MYB* 3′-UTR in HEK-293T cells overexpressing *miR-187-5p*. The HEK-293T cells were transfected with *miR-187-5p* mimics, firefly luciferase plasmids, and Renilla luciferase vectors. The relative activity of firefly luciferase was measured and normalised to that of Renilla luciferase. The data are presented as mean ± SD (*n* = 3). *p < 0.05.

qRT-PCR and western blotting were used to investigate the mechanism underlying the influence of *miR-187-5p* on cellular function. The mRNA and protein levels of MYB were significantly downregulated in the LA-treated groups (Figures 3A,B). The levels of *MYB* mRNA were significantly reduced when *miR-187-5p* was overexpressed (Figure 3C). Using bioinformatics analysis, we validated *MYB* as a target gene of *miR-187-5p*; two binding sites (2,710–2,732 nt and 3,634–3,656 nt) were predicted in the 3′-UTR of *MYB*, which were complementary to the seed sequence of *miR-187-5p* (Figure 3D). The direct interactions between *miR-187-5p* and *MYB* were examined using luciferase reporter plasmids containing the wild-type *MYB* (*MYB*-wt) and binding site mutants (*MYB*-site1-mut, *MYB*-site2-mut, and *MYB*-site1+2-mut). The luciferase activity of *MYB*-wt decreased significantly, with the transfection of *miR-187-5p* mimics; however, they had no influence on *MYB*-site1, *MYB*-site2, and *MYB-*site1+2 (Figure 3E), indicating that *MYB* is a direct target gene of *miR-187-5p*. The LAs, lidocaine and bupivacaine, upregulated the expression of *miR-187-5p*, directly downregulating the expression of *MYB*.

### *DANCR* Decoys *miR-187-5p* by Reciprocal Suppression

**Figure 4:**
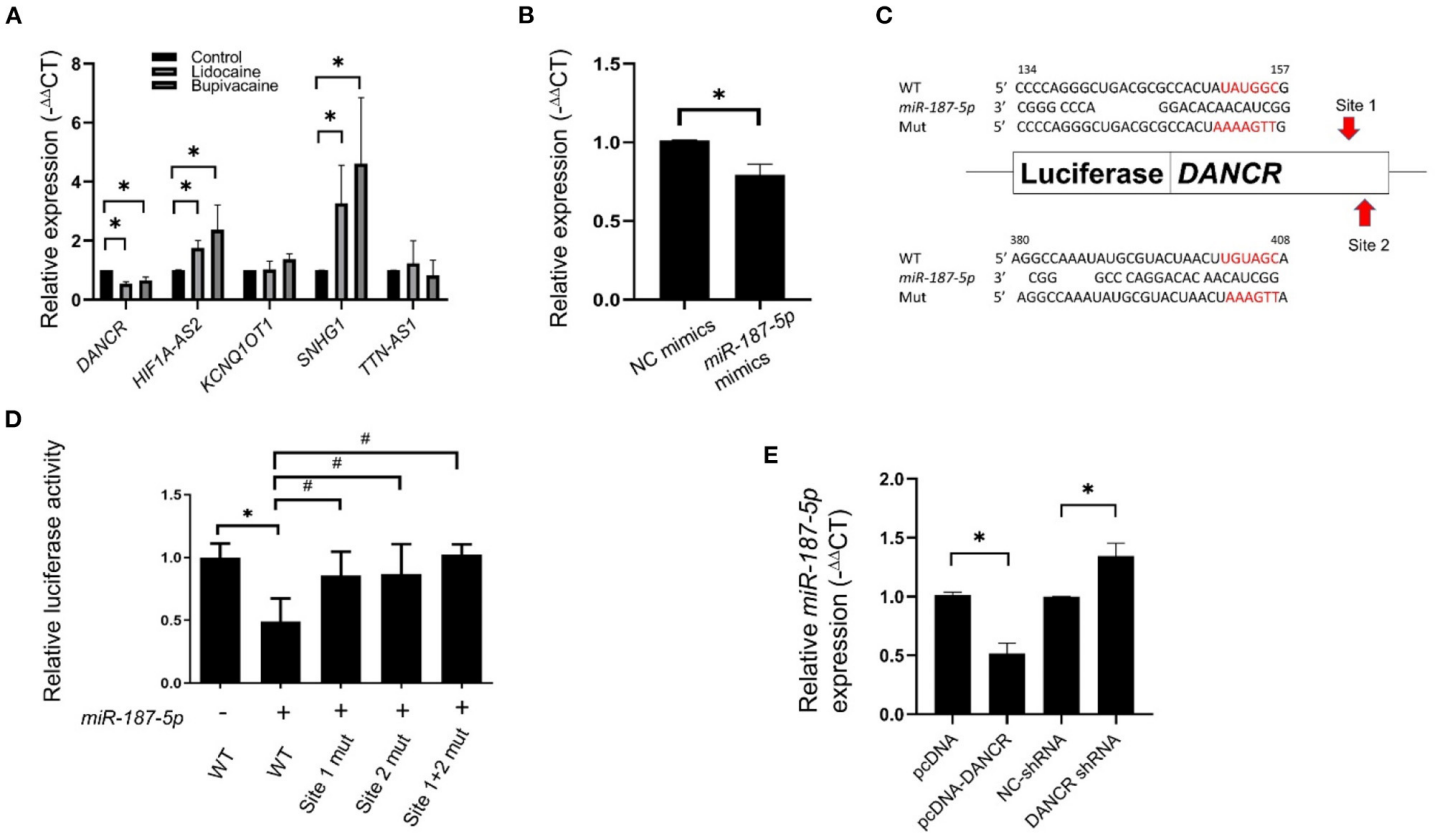
Expression of *DANCR* lncRNA was downregulated in MCF-7 cells following treatment with lidocaine or bupivacaine. **(A)** The relative expression levels of selective lncRNAs in MCF-7 cells following treatment with lidocaine (4 mM) or bupivacaine (1 mM). The expression levels were measured using qRT-PCR and normalised to that of 18S rRNA. **(B)** Relative expression levels of *DANCR* in MCF-7 cells overexpressing *miR-187-5p*. The cells were transfected with 2 μg of *miR-187-5p* or control mimics. The expression levels were detected using qRT-PCR and normalised to that of 18S rRNA. **(C)** Schematic diagram of the putative binding site of *miR-187-5p* in the binding region of *DANCR*. **(D)** Luciferase reporter assays of *DANCR* in HEK-293T cells overexpressing *miR-187-5p*. The HEK-293T cells were transfected with *miR-187-5p* mimics, firefly luciferase plasmids, and Renilla luciferase vectors. The relative activity of firefly luciferase was measured and normalised to that of Renilla luciferase. **(E)** The relative expression levels of *miR-187-5p* in MCF-7 cells overexpressing *DANCR* or shRNAs against *DANCR*. The expression levels were detected using qRT-PCR and normalised to that of U6 snRNA. The data are presented as mean ± SD (*n* = 3). *p < 0.05.

LncRNAs act as ceRNAs and influence mRNA levels by sequestering miRNAs that target both lncRNA and mRNAs. To identify the ceRNAs of *miR-187-5p*, we used DIANA-LncBase v2 (23) for predicting the lncRNAs that bind to *miR-187-5p*. Five possible lncRNAs were predicted and validated using qRT-PCR (Figure 4A). The expression of *DANCR* was downregulated by the LAs, while that of the *HIF1A-AS2* and *SNHG1* lncRNAs was upregulated; however, these lncRNAs did not function as ceRNAs of *miR-187-5p*. In addition, the expression of *DANCR* significantly decreased when the expression of *miR-187-5p* increased (Figure 4B).

Luciferase reporter assays were performed for validating the association between *DANCR* and *miR-187-5p*. Prediction revealed two potential binding sites of *miR-187-5p* at 134–157 nt and 380–408 nt of *DANCR*, which were individually positioned upstream of the luciferase reporter genes (Figure 4C). The binding site mutants (sites 1-mut, 2-mut, and 1+2-mut) were constructed and transfected into HEK-293T cells. The luciferase activity of *DANCR*-wt was significantly reduced by the *miR-187-5p* mimics; however, they had no effect on sites 1-mut, 2-mut, and 1+2-mut (Figure 4D), indicating that the *DANCR* lncRNA sponges *miR-187-5p* by direct binding.

The overexpression of *DANCR* suppressed *miR-187-5p* expression, while the downregulation of *DANCR* increased *miR-187-5p* expression (Figure 4E). These results demonstrated a reciprocal inhibitory relationship between *DANCR* and *miR-187-5p*, and that *miR-187-5p* could mediate the inhibitory effect of the LAs on MCF-7 cell viability and migration.

## Discussion

The LAs, lidocaine and bupivacaine, inhibited MCF-7 cell proliferation and migration. Genomic studies and validation experiments revealed that the LAs promoted *miR-187-5p* expression, which downregulated *MYB* expression. Additionally, the LAs reduced the expression of *DANCR* lncRNA, which could be a ceRNA of *miR-187-5p*. Our study demonstrated a novel mechanism underlying the inhibitory effect of LAs on cancer cell proliferation, migration, and invasion, mediated via the *DANCR*-*miR-187-5p*-*MYB* regulatory axis.

LAs inhibit cancer cell proliferation, migration, and invasion by regulating miRNA expression (12, 13). Lidocaine inhibits proliferation and induces apoptosis in retinoblastoma cells by modulating the *miR-520a-3p*/EGFR axis (24). Bupivacaine inhibits gastric cancer progression by regulating the *circ_0000376*/*miR-145-5p* axis (25). Using NGS and qRT-PCR, we demonstrated that lidocaine and bupivacaine upregulated *miR-187-5p* expression. *MiR-187-5p* regulates cellular proliferation and apoptosis in lung cancer (26), hepatocellular carcinoma (27), bladder cancer (28), and acute lymphoblastic leukaemia (29), and is a possible indicator of drug sensitivity in breast cancer (30). This study is the first to identify that *miR-187-5p* regulates the inhibitory effect of LAs on the proliferation, migration, and invasion of MCF-7 cells.

Bioinformatics-based prediction revealed that *MYB* is a target gene of *miR-187-5p*, consistent with the reports that *MYB* functions as an oncogene in different tumours, including breast cancer (21, 31–33). *MYB* could be inhibited in MCF-7 cells through the upregulation of *miR-187-5p* by lidocaine or bupivacaine. These LAs reduced the mRNA and protein levels of MYB, and the *miR-187-5p* mimics significantly reduced *MYB* expression in MCF-7 cells. The luciferase reporter assay confirmed that *miR-187-5p* directly targets the 3′-UTR of *MYB*.

**Figure 5:**
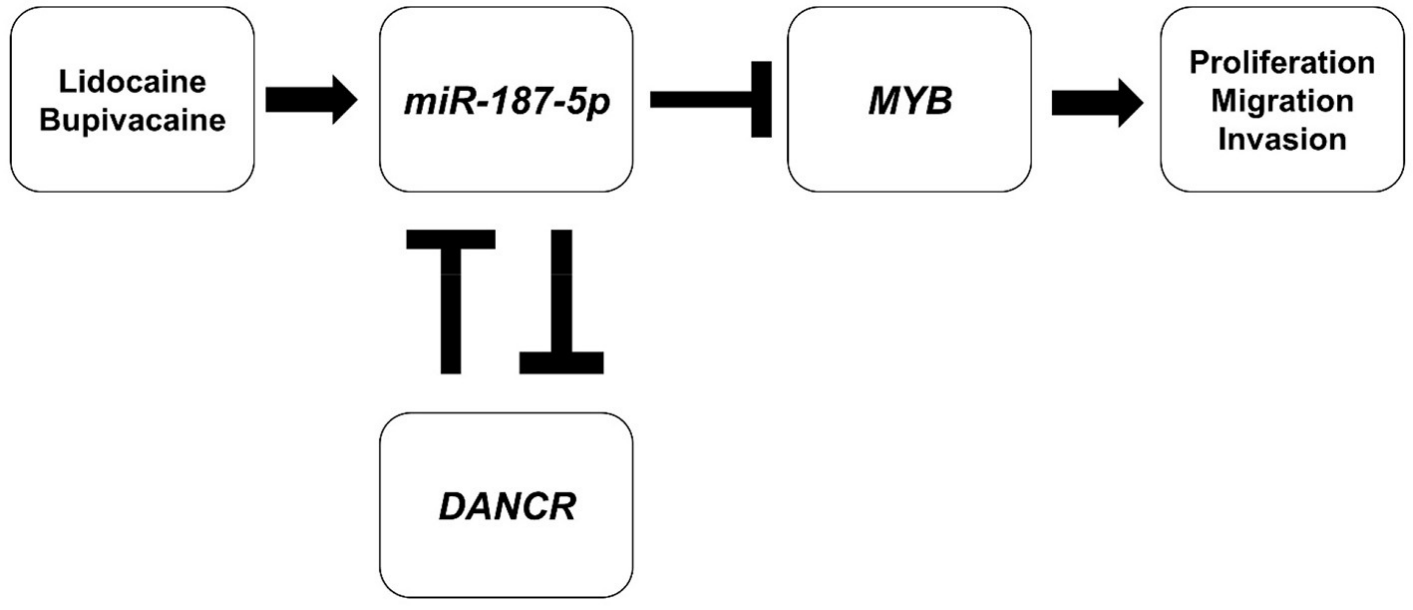
Schematic diagram of the proposed mechanism underlying the inhibitory effect of LAs. Lidocaine and bupivacaine suppress the malignancy of MCF-7 breast cancer cells by upregulating *miR-187-5p*, which in turn downregulates the expression of MYB (MYB proto-oncogene, transcription factor) proteins and the DANCR (Differentiation Antagonising Non-Protein Coding RNA) lncRNA.

LncRNAs interact with various molecules and have key roles in regulating signalling processes at the epigenetic, transcriptional, and post-transcriptional levels (34, 35). A novel regulatory mechanism elucidates that all types of transcripts, including mRNAs, lncRNAs, and circular RNAs, can act as ceRNAs, forming a complex RNA interactome involving different RNA species, for regulating gene expression (35). The lncRNA-miRNA-mRNA crosstalk could have a prominent role in the anti-tumour effects of LAs. Among the lncRNAs, *DANCR*, encoded by a gene located in human chromosome 4q12, plays an important role in different cancers (36). The *DANCR*-SOCS3-EZH2 axis regulates the inflammatory phenotype and breast cancer cell metastasis (37). *DANCR* promotes progressive osteosarcoma by functioning as a ceRNA and sponging *miR-335-5p* and *miR-1972*, regulating ROCK1 expression (38). In this study, the expression of *DANCR* was inhibited by the LAs, and a reciprocal suppressive effect was observed between *DANCR* and *miR-187-5p*. The luciferase reporter assay confirmed that *DANCR* was the target of *miR-187-5p*. Therefore, the upregulation of *miR-187-5p* and downregulation of *DANCR* could mediate the inhibitory effect of LAs on the proliferation, migration, and invasion of MCF-7 cells. Together, we concluded that the DANCR-miR-187-5p-MYB axis is activated by lidocaine and bupivacaine; the proposed schematic diagram is presented in Figure 5.

This study has certain limitations. First, LAs are chemically categorised into amide-ester and amide-amide groups, and the new generation LAs, including ropivacaine and levobupivacaine, are used clinically. Only the older generation LAs, lidocaine and bupivacaine, were considered. LAs have variable anti-tumour effects and mechanisms of action in different cancers (39). Further studies are necessary for confirming whether the *DANCR*-*miR-187-5p*-*MYB* axis mediates the inhibitory effects of other LAs. Second, there are several breast cancer types; and therefore, further studies are necessary for confirming the effects of lidocaine and bupivacaine in other breast cancer cell lines and in an animal model, which may improve upon the present findings. Third, although luciferase studies proved the interaction between *DANCR-miR-187-5p-MYB* axis, whether the anti-tumour properties of LAs can be reversed requires more experiments to prove, such as using antagomir to inhibit *miR-187-5p* or overexpressing *MYB* in MCF-7 cells treated with LAs.

This study demonstrated that lidocaine and bupivacaine inhibited the growth and metastasis of breast cancer cells. The anti-tumour properties of these LAs were partially attributed to the upregulation of *miR-187-5p*, which inhibited *MYB* signalling by directly binding to *MYB*. Screening for the possible targets of *miR-187-5p* using a dual-luciferase reporter assay revealed that the *DANCR* lncRNA directly targets *miR-187-5p*, suggesting that *DANCR* could act as a *miR-187-5p* sponge. In summary, we report that the *DANCR*-*miR-187-5p*-*MYB* axis may be activated by lidocaine and bupivacaine.

## Data Availability Statement

The original contributions presented in the study are publicly available. This data can be found at: https://www.ncbi.nlm.nih.gov/geo/, GSE171282.

## Author Contributions

C-YL and L-CL: conception and design of experiments and manuscript preparation. C-YL and W-TT: performed experiments. C-YL and Y-YC: data analyses. L-CL, M-HT, and EC: contributed reagents, materials, and analytical tools. All the authors reviewed and approved the final version of the manuscript.

## Funding

This work was supported by a grant from the Ministry of Science and Technology [MOST 109-2320-B-002-016-MY3]. These funding sources had no role in the design of this study and will not have any role during its execution, analyses, interpretation of the data, or decision to submit results.

## Conflict of Interest

The authors declare that the research was conducted in the absence of any commercial or financial relationships that could be construed as a potential conflict of interest.

## Publisher's Note

All claims expressed in this article are solely those of the authors and do not necessarily represent those of their affiliated organizations, or those of the publisher, the editors and the reviewers. Any product that may be evaluated in this article, or claim that may be made by its manufacturer, is not guaranteed or endorsed by the publisher.

## Abbreviations

LA: local anaesthetic
ncRNA: non-coding RNA
miRNA: microRNA
PCR: polymerase chain reaction
sncRNA: small ncRNA
lncRNA: long ncRNA
EMT: epithelial-mesenchymal transition
ceRNA: competing endogenous RNA
MRE: miRNA response element
RISC: RNA-induced silencing complex
MYB, MYB proto-oncogene: transcription factor
*DANCR*: Differentiation Antagonising Non-Protein Coding RNA
qRT-PCR: quantitative real time polymerase chain reaction
PCA: principal component analysis.
