# Supplementary material for: Lidocaine and Bupivacaine Downregulate MYB and DANCR lncRNA by Upregulating miR-187-5p in MCF-7 Cells
Source: Front Med (Lausanne). 2022 Jan 13;8:732817. doi: 10.3389/fmed.2021.732817 (PMC8792760; doi:10.3389/fmed.2021.732817)
Supplement: Supplementary file 1 [file Table_1.DOCX]

| **Supplementary Table 1. Primers used for reverse transcription and quantitative Real Time-polymerase chain reaction (qRT-PCR)** | |
| --- | --- |
| Gene transcript | Primer sequence |
| **Reverse Transcription** | |
| *miR-99a-3p* | 5′-GTTGGCTCTGGTGCAGGGTCCGAGGTATTCGCACCAGA  GCCAACCAGACC-3′ |
| *miR-187-5p* | 5′-GTTGGCTCTGGTGCAGGGTCCGAGGTATTCGCACCAGA  GCCAACGCCCGG-3′ |
| *miR-197-5p* | 5′-GTTGGCTCTGGTGCAGGGTCCGAGGTATTCGCACCAGA  GCCAACCCTCCC-3′ |
| *miR-223-5p* | 5′-GTTGGCTCTGGTGCAGGGTCCGAGGTATTCGCACCAGA  5′-GCCAACAACTCA-3′ |
| *miR-363-5p* | 5′-GTTGGCTCTGGTGCAGGGTCCGAGGTATTCGCACCAGA  GCCAACAAATTG-3′ |
| *miR-377-5p* | 5′-GTTGGCTCTGGTGCAGGGTCCGAGGTATTCGCACCAGA  GCCAACGAATTC-3′ |
| *miR-515-5p* | 5′-GTTGGCTCTGGTGCAGGGTCCGAGGTATTCGCACCAGA  GCCAACCAGAAA-3′ |
| *miR-3150-5p* | 5′-GTTGGCTCTGGTGCAGGGTCCGAGGTATTCGCACCAGA  GCCAACGCTGGG-3′ |
| *miR-3162-3p* | 5′-GTTGGCTCTGGTGCAGGGTCCGAGGTATTCGCACCAGA  GCCAACTGGGGA-3′ |
| *miR-5702* | 5′-GTTGGCTCTGGTGCAGGGTCCGAGGTATTCGCACCAGA  GCCAACCATGGG-3′ |
| *miR-6830-3p* | 5′-GTTGGCTCTGGTGCAGGGTCCGAGGTATTCGCACCAGA  GCCAACCTGCAA-3′ |
| U6 snRNA | 5′-CGCTTCACGAATTTGCGTGTCAT-3′ |
| **Quantitative RT-PCR** | |
| *MYB*  *(NM_001130173.2)* | Forward 5′-GAAAGCGTCACTTGGGGAAAA-3′ |
|  | Reverse 3′-TGTTCGATTCGGGAGATAATTGG-5′ |
| *DANCR*  *(NR_024031.2)* | Forward 5′-CTGCATTCCTGAACCGTTATCT-3′ |
|  | Reverse 3′-GGGTGTAATCCACGTTTCTCAT-5′ |
| *HIF1A-AS2*  *(NR_045406.1)* | Forward 5′-AAAGCTTGGGCAAATTATTCA-3′ |
|  | Reverse 3′-TGAATGGGATGAGTGAAGCA-5′ |
| *KCNQ1OT1*  *(NR_002728.3)* | Forward 5′-TGCAGAAGACAGGACACTGG-3′ |
|  | Reverse 3′-CTTTGGTGGGAAAGGACAGA-5′ |
| *SNHG1*  *(NR_003098.2)* | Forward 5′-GCCAGCACCTTCTCTCTAAAGC-3′ |
|  | Reverse 3′-GTCCTCCAAGACAGATTCCATTTT-5′ |
| *TTN-AS1*  *(NR_038272.1)* | Forward 5′-TTAGCGCAGCTCTCCTTCAC-3′ |
|  | Reverse 3′-AAGCAACACCGCAGTTCCAT-5′ |
| *miR-99a-3p* | Forward 5′-GCGGCGGCAAGCTCGCTTCTAT-3′ |
|  | Reverse 3′-GTGCAGGGTCCGAGGT-5′ |
| *miR-187-5p* | Forward 5′-GGCTACAACACAGGACCCGGGC-3′ |
|  | Reverse 3′-GTGCAGGGTCCGAGGT-5′ |
| *miR-197-5p* | Forward 5′-CGGGTAGAGAGGGCAGTGGGAGG-3′ |
|  | Reverse 3′-GTGCAGGGTCCGAGGT-5′ |
| *miR-223-5p* | Forward 5′-CGTGTATTTGACAAGCTGAGTT-3′ |
|  | Reverse 3′-GTGCAGGGTCCGAGGT-5′ |
| *miR-363-5p* | Forward 5′-GCGGCGGCGGGTGGATCACGATG-3′ |
|  | Reverse 3′-GTGCAGGGTCCGAGGT-5′ |
| *miR-377-5p* | Forward 5′-AGAGGTTGCCCTTGGTGAATTC-3′ |
|  | Reverse 3′-GTGCAGGGTCCGAGGT-5′ |
| *miR-515-5p* | Forward 5′-TTCTCCAAAAGAAAGCACTTTCTG-3′ |
|  | Reverse 3′-GTGCAGGGTCCGAGGT-5′ |
| *miR-3150b-5p* | Forward 5′-GCGGCGGCAACCTCGAGGATCTC-3′ |
|  | Reverse 3′-GTGCAGGGTCCGAGGT-5′ |
| *miR-3162-3p* | Forward 5′-GCGGCGGTCCCTACCCCTCCAC-3′ |
|  | Reverse 3′-GTGCAGGGTCCGAGGT-5′ |
| *miR-5702* | Forward 5′-GCGGCGGTGAGTCAGCAACATAT-3′ |
|  | Reverse 3′-GTGCAGGGTCCGAGGT-5′ |
| *miR-6830-3p* | Forward 5′-GCGGCGGTGTCTTTCTTCTCTCCC-3′ |
|  | Reverse 3′-GTGCAGGGTCCGAGGT-5′ |
| *U6* snRNA | Forward 5′-GCTTCGGCAGCACATATACTAAAAT-3′ |
|  | Reverse 3′-CGCTTCACGAATTTGCGTGTCAT-5′ |
| *ACTB* | Forward 5′-GGGAAATCGTGCGTGAC-3′ |
|  | Reverse 3′-CAAGAAGGAAGGCTGGAA-5′ |
| 18s rRNA | Forward 5′-TCAACTTTCGATGGTAGTCGCCGT-3′ |
|  | Reverse 3′-TCCTTGGATGTGGTAGCCGTTTCT-5′ |
